# Gut-Directed Hypnotherapy: An Option to Enhance the Biopsychosocial Treatment of Disorders of Gut–Brain Interaction

**DOI:** 10.3390/brainsci16101029

**Published:** 2026-09-26

**Authors:** Peter J. Whorwell

**Affiliations:** Neurogastroenterology Unit, Wythenshawe Hospital, Southmoor Road, Manchester M23 9LT, UK; peter.whorwell@mft.nhs.uk

**Keywords:** irritable bowel syndrome, treatment, hypnotherapy, biopsychosocial

## Abstract

**Highlights:**

**What are the main findings?**

- Hypnotherapy focused on the gut can significantly improve the symptoms of irritable bowel syndrome and related conditions.
- Gut-directed hypnotherapy is not a stand-alone treatment but should form part of an individualised package of treatment options.

**What are the implications of the main findings?**

- This form of hypnotherapy could improve the lives of many patients who had previously failed to respond to treatment.
- The beneficial effects of gut-directed hypnotherapy are long-lasting.

**Abstract:**

The most common gastrointestinal disorders encountered in secondary care are the functional gastrointestinal disorders, now known as disorders of gut brain interaction (DGBI) of which irritable bowel syndrome (IBS) is the most prevalent example. The pathophysiology of these conditions is beginning to be better understood although they are likely to have a multifactorial basis rather than there being a single ‘cause’. The biopsychosocial treatment model recommends that rather than just relying on pharmacology, everything about an individual’s situation should be considered in their management including psychological and social factors. Gut directed hypnotherapy (GDH) has been shown to reduce symptoms, decrease psychological distress, improve quality of life and normalise the physiology of the gut in patients with IBS. It can also be used to explore the psychosocial issues affecting an individual and provides an opportunity to enhance a biopsychosocial approach to treating a range of gastrointestinal disorders. We have been researching GDH for IBS and related disorders for many years and some of the emerging evidence has now reached a point where, for instance, it is now endorsed for the treatment of IBS in a number of national and international guidelines.

Irritable bowel syndrome (IBS) and related conditions, previously referred to as ‘functional gastrointestinal disorders’, have recently been re-categorised as ‘disorders of gut–brain interaction (DGBI)’ in an attempt to reflect the importance of the combined role of the gut and the central nervous system in their pathophysiology. However, even this change in nomenclature does not reflect the true complexity of these conditions.

IBS is the most common DGBI affecting approximately 10% of the population and accounting for at least a third of a gastroenterologist’s workload. The principal symptoms are abdominal pain, abdominal bloating and bowel dysfunction which can take the form of constipation, diarrhoea or an alternation between the two [1]. In addition, patients often complain of one or more ‘non-colonic’ symptoms such as lethargy, low backache, nausea, chest pain and bladder symptoms [2] which can be just as intrusive as the bowel symptoms [3]. Originally, it was considered that IBS was just a psychosomatic problem, the symptoms of which were caused by stress leading to spasm and disordered motility of the smooth muscle of the colon. Consequently, the principal treatment option was the use of antispasmodics in combination with a diet high in insoluble fibre and either laxatives or antidiarrhoeals where appropriate. This approach still helps some patients, particularly those with relatively mild symptoms, but many individuals do not respond and often get worse over time. They continue to have multiple consultations, repetitive investigations and fail to respond to treatment which contributes to IBS gaining a reputation for being notoriously difficult to manage. These refractory IBS sufferers are often referred to tertiary care specialist centres where the women liken the pain to that of childbirth and 57% report faecal incontinence irrespective of whether they have diarrhoea or constipation [4]. It has been shown that 38% of these patients admit to suicidal ideation which is related to hopelessness about the prospect of finding a solution to their symptoms rather than due to depression [5]. It is, therefore, perhaps not surprising that IBS imposes a significant burden on healthcare resources and it has been estimated that the direct costs to the UK National Health Service are between £1 and £2 billion annually [6]. These observations, which are not just confined to IBS, highlight the need to find better ways of managing DGBIs.

As time has gone by it has become apparent that the pathophysiology of IBS is far more complicated than it just being a motility disorder. A whole range of other factors have now been implicated, including abnormalities of visceral sensation, disturbed brain processing of noxious stimuli from the gut, low-grade inflammation, bacterial imbalance (dysbiosis), neuroendocrine influences, dietary factors and psychosocial inputs [7,8]. The ‘doses’ of these factors probably vary from patient to patient and are likely to be cumulative until they reach a threshold where they cause problems. Furthermore, if the ‘dose’ of one on its own is sufficiently large, it may be able to cause trouble on its own. To complicate matters even more, it is also now recognised that rather than just involving the colon, patients with IBS have symptoms referable to the whole gastrointestinal tract including the stomach and oesophagus. It therefore follows that with such a complex multifactorial disease process, treatment is going to be challenging in the absence of one specific target to which a pharmacological agent can be directed.

Consequently, the pharmacological treatment of IBS usually consists of a process of ‘trial and error’ using medications that might improve symptoms either directly (for example, laxatives, antidiarrhoeals and antispasmodics) or by targeting one or more of the putative causative factors [9]. Many years ago, it was shown that a tricyclic antidepressant in combination with fluphenazine, mebeverine and ispaghula improved the symptoms of IBS [10]. This has resulted in a number of subsequent studies confirming that antidepressants, particularly those of the tricyclic variety, have beneficial effects in IBS even when used at low doses that would not normally be expected to improve depression [11]. This observation suggests that these agents exert their benefits by a different mechanism to their antidepressant activity such as reducing visceral hypersensitivity [12] and has led to the concept of neuromodulation. This is a particularly useful term when using these medications in IBS as it helps to convince the patients that we are not using these medications just because we think they are depressed.

Many patients with IBS say that eating makes their symptoms worse [13,14] to the extent that some individuals resort to very restricted diets and there seem to be two main reasons for this finding. The first is likely to be a specific intolerance to one or more foods and the second is a possible exaggerated cephalic response to eating, making the act of eating itself, rather than the food, the source of the problem. This cephalic phase has been elegantly demonstrated in a study showing that talking about food, smelling food, or chewing but not swallowing food (sham feeding) can all stimulate the colon [15]. Patients with IBS tend to have a higher prevalence of eczema, asthma and hay fever than the general population and it therefore seems reasonable to assume that, at least in some of these individuals, an allergic reaction to food might be resulting in symptoms [16]. Consequently, it is rather surprising that this possibility has received relatively little research attention although the empirical use of antihistamines or mast cell stabilisers is worth considering. Needless to say, a whole range of diets have been advocated for use in IBS with the exclusion of FODMAPs (fermentable oligosaccharides, disaccharides, monosaccharides and polyols) being one that is most used and has strong supporting evidence [17], although reducing fibre intake, particularly of the insoluble variety, can also be helpful [18].

Some DGBI patients have symptoms suggestive of dysfunction in other anatomical areas of the gastrointestinal system such as the oesophagus, stomach or even the biliary system. In certain individuals, these symptoms can be so predominant that they do not even complain of symptoms that are compatible with a diagnosis of IBS. These observations have led the Rome Foundation to propose a classification of disorders of gut–brain interaction based on the anatomical site from which the patient’s symptoms are thought to have most likely originated, such as non-cardiac chest pain, functional dyspepsia, cyclical vomiting syndrome, sphincter of Oddi dysfunction and proctalgia fugax [19]. The management of these conditions shares many of the principles used in IBS in conjunction with additional pharmacological approaches targeting specific symptoms associated with these problems, for instance, the use of antiemetics or acid suppression.

Most medical specialties will have experience of a least one ‘functional’ disorder affecting the system in which they are interested, such as fibromyalgia in rheumatology, functional neurological disorder in neurology, pelvic pain in gynaecology and overactive bladder in urology. Clinical experience suggests there is considerable overlap in these conditions between specialties, but this possibly is not quite so well-recognised in modern specialty medicine where training and practice is so heavily concentrated in the particular specialty concerned. This can lead to a patient with a ‘functional’ disorder in one system being referred to the wrong specialty because of associated symptoms which have not be recognised as being part of their functional disorder syndrome. For instance, IBS has been shown to be over-represented in gynaecological and urological outpatients as well as in care of the elderly clinics. Unfortunately, in this situation unnecessary investigation and treatment often takes place resulting in poor outcomes and patient dissatisfaction [20,21,22,23].

In the past, these ‘functional’ disorder patients were often referred to as individuals with ‘medically unexplained symptoms’ and a common feature of all of them is that they are very refractory to treatment. This is not surprising as there is often an absence of a specific pharmacological target. It is in this group of disorders that the biopsychosocial approach to treatment, which was proposed by Dr George Engel [24] in the 1970s, would seem to be most appropriate way of managing these patients and IBS is no exception.

When considering IBS, the ‘bio’ component of treatment is well recognised and, as already described, involves antispasmodics, laxatives, anitidiarrhoeals and neuromodulators amongst other medications. Hypnotherapy provides a way of enhancing the psychosocial approach as not only does it offer psychological support, but a session also provides an opportunity to explore any other issues affecting the individual, including their social situation. It is amazing how much more the therapist learns about a patient during their session than a doctor in a busy gastroenterology clinic. In addition, hypnosis can be used to help individuals control physiological functions not immediately considered to be under conscious control, so this might also actually be able to complement the ‘bio’ component of treatment.

Consequently, in the early 1980s we started to explore the possibility that hypnotherapy might have a role in the treatment of IBS. We developed a technique called gut-directed hypnotherapy (GDH) where the emphasis is on normalising the physiological abnormalities associated with IBS although psychosocial issues are also addressed during sessions. In our first trial, IBS patients were randomised to receive either a course of GDH or a control treatment of a similar duration consisting of supportive listening combined with a placebo medication [25]. Patients receiving GDH experienced a greater reduction in symptoms and improvement in well-being than those patients allocated to control treatment. These encouraging results have been confirmed in subsequent studies by other investigators [26,27,28,29,30] and in a large audit of one thousand consecutive IBS patients treated in our unit 76% (80% females, 62% males) reported a significant response to treatment [31]. Responders experienced a reduction in IBS symptoms and non-colonic symptoms as well as an improvement in scores for abdominal pain, quality of life, anxiety and depression. GDH is also used in children where it is just as effective as it is in adults [32,33,34]. As a department that sees patients from all over the UK, we introduced video hypnotherapy long before the pandemic and have shown that it does not appear to be inferior to face-to-face treatment [35]. In addition, it is very useful for patients who have difficulty travelling even short distances because of the severity of their symptoms, especially diarrhoea. The benefits of hypnotherapy are not just confined to IBS, and we have shown that other DGBIs such as functional dyspepsia [36] and non-cardiac chest pain [37] are equally responsive to this form of treatment. Importantly, the beneficial effects of GDH are long-lasting [33,36,38,39].

This improvement in DGBI symptoms is associated with the additional capacity of hypnosis to influence a variety of physiological processes such as gastrointestinal motility [40,41], visceral sensitivity [42,43,44], the response of the brain to painful peripheral stimuli [45], the gastrocolonic reflex [46] and gastric acid secretion [47,48]. Although there has been no research on the effect of hypnotherapy on the putative inflammatory component of IBS, there is some preliminary evidence that hypnotherapy might have a beneficial effect in inflammatory bowel disease and the immunological processes involved in its pathogenesis [49,50,51,52].

In conclusion, there is now good evidence that hypnotherapy is very effective in reducing symptoms and modifying the physiological abnormalities associated with DGBIs as well as reducing consultation behaviour, medication needs and absenteeism from work [53]. However, it should not be regarded as a standalone treatment. It is far better to apply it as part of a package of measures which include patient education, ongoing support, dietary manipulation and traditional pharmacology. Medications such as laxatives, antidiarrhoeals or antispasmodics may still be needed although often less frequently or at lower doses and the diet might still need to be somewhat restricted. GDH is very safe with minimal side effects and can be used to treat IBS of any severity although it is most cost-effective in refractory patients who are heavy users of healthcare resources. For gastroenterology departments offering this form of treatment without the availability of psychiatric support, it is best to avoid patients with severe psychiatric comorbidities and it is contraindicated in those with dissociative disorders. Once a course of treatment is completed, patients are strongly encouraged to continue practicing self-hypnosis with or without the aid of an audio recording, and some individuals may need the occasional ‘top-up’ session from a therapist.

Unfortunately, the provision of GDH in the UK is very limited despite the fact that it was developed in this country. Group hypnotherapy [28,54,55,56] or hypnotherapy apps [57,58] may help to improve provision although the evidence so far suggests compliance and efficacy may not be quite as good as face-to-face or video treatment, especially for the more severe forms of IBS and related disorders. There is a need for the provision of GDH to meet certain standards in terms of level of training of therapists, the use of protocols known to be effective and the recording of outcomes using standard measures. To help improve the supply of suitably trained hypnotherapists for the UK National Health Service, we have now established an annual training programme for healthcare professionals such as nurses, dieticians and physiotherapists who are particularly suited to providing this form of treatment to patients with these gastrointestinal disorders.

## Data Availability

No new data were created or analysed in this study. Data sharing is not applicable to this article.
